# Effect of carboxylesterase 1 S75N on clopidogrel therapy among acute coronary syndrome patients

**DOI:** 10.1038/s41598-017-07736-1

**Published:** 2017-08-03

**Authors:** Fei-Yan Xiao, Jian-Quan Luo, Min Liu, Bi-Lian Chen, Shan Cao, Zhao-Qian Liu, Hong-Hao Zhou, Gan Zhou, Wei Zhang

**Affiliations:** 1 Department of Clinical Pharmacology, Xiangya Hospital, Central South University, Changsha, 410008 China; 2 Institute of Clinical Pharmacology, Central South University; Hunan Key Laboratory of Pharmacogenetics, Changsha, China; 3 National institution of drug clinical trial, Xiangya Hospital, Central South University, Changsha, China; 4 Department of cardiovascular, Zhengzhou central hospital, Zhengzhou University, Zhengzhou, China; 5 Department of Geriatrics, Xiangya Hospital, Central South University, Changsha, China

## Abstract

Carboxylesterase 1 (*CES1*) hydrolyzes the prodrug clopidogrel to an inactive carboxylic acid metabolite. The effects of *CES1* S75N (rs2307240,C>T) on clopidogrel response among 851 acute coronary syndrome patients who came from the north, central and south of China were studied. The occurrence ratios of each endpoint in the CC group were significantly higher than in the CT + TT group for cerebrovascular events (14% vs 4.8%, p < 0.001, OR = 0.31), acute myocardial infarction (15.1% vs 6.1%, p < 0.001, OR = 0.37) and unstable angina (62.8% vs 37.7%, p < 0.001, OR = 0.36). The results showed that there was a significant association between *CES1* S75N (rs2307240) and the outcome of clopidogrel therapy. Moreover, the frequency of the T allele of rs2307240 in acute coronary syndrome patients (MAF = 0.22) was more than four times higher than that in the general public (MAF = 0.05).

## Introduction

Dual antiplatelet therapy (DAPT), a combination treatment of clopidogrel and aspirin, is the standard therapy for preventing recurrent cardiovascular events in patients who have coronary heart disease (CHD), who undergo a percutaneous coronary intervention (PCI) and who have acute coronary syndrome (ACS)^1^. It has been reported that the efficacy of clopidogrel treatment varies from person to person; 5–40% of patients do not receive therapeutic benefit and instead have an increased risk of adverse outcomes^2^. Some patients experience acute myocardial infarction, unstable angina, stent thrombosis (ST) and/or cerebrovascular events. Clinical, genetic, and cellular factors might contribute to the variability in clopidogrel response, and genetic factors have been estimated to explain approximately 70% of the inter-individual variance in clopidogrel pharmacokinetics and pharmacodynamics acute myocardial infarctions^3^.

Clopidogrel is a thienopyridine prodrug that is converted to a pharmacologically active thiol metabolite through the inactive intermediate 2-oxo-clopidogrel. This reaction is catalyzed by cytochrome P450 (CYP) enzymes, including CYP2C19, CYP3A, CYP2B6, CYP1A2, and CYP2C9. However, only 15% of a clopidogrel dose is available from this pathway. The 5-thiol clopidogrel active metabolite inhibits ADP-induced platelet activation and aggregation by irreversibly binding to the P2Y12 receptor on the surface of platelets^4, 5^. Approximately 85% of the parent clopidogrel is rapidly hydrolyzed to the inactive metabolite, clopidogrel carboxylic acid, which is further metabolized by glucuronidation^6^. This hydrolysis is catalyzed by hepatic carboxylesterase 1 (CES1)^7^, which is primarily expressed in the liver^8^. In addition, CES1 can hydrolyze 2-oxo-clopidogrel and 5-thiol clopidogrel active metabolites to 2-oxo-clopidogrel carboxylate^9^, an inactive metabolite, and the 5-thiol carboxylic acid metabolite. Ultimately, only a small proportion of clopidogrel is converted to the active metabolite^10^.

In humans, CES1 is a widely expressed serine esterase, catalyzing the hydrolysis of multiple amide and ester-containing endogenous compounds, toxins, and medications to their respective free acids^11^. CES1 is involved in most hydrolytic activities in the human liver. Significant inter-individual variability of *CES1* expression and/or activity has been consistently reported in the bio-medical literature. This variability is likely the result of both genetic and environmental factors^12, 13^. The genetic variants involved in the pharmacokinetics of clopidogrel have been investigated extensively; A previous study documented that the *CES1* single nucleotide polymorphisms (SNPs) G143E^14, 15^ (rs71647871) and D260fs^16^ (rs71647872) exhibited markedly decreased enzymatic activity in the hydrolysis of the CES1 substrate methylphenidate and *CES1A2* −816C was associated with attenuated platelet reactivity to clopidogrel^17^. According to a previous report, the metabolic activity of CES1A1 can differ up to 31-fold between individuals^18^. Therefore, it has been hypothesized that *CES1* genetic variants could serve as biomarkers to predict clopidogrel response and individualize clopidogrel dosing regimens in clinical practice^19^.

In this study, we investigated whether *CES1* S75N could significantly influence the efficacy of clopidogrel and alter the patients’ response.

## Results

### Genotyping and frequencies of *CES1* S75N in Chinese acute coronary syndrome patients

Patients with acute coronary syndrome who were taking a standard loading or maintenance clopidogrel regime were recruited. The characteristics of the study participants can be summarized as follows. Generally, the patients were older (mean age = 63.25 ± 11.79 years) and had a high prevalence of hypertension (69.1%), hyperlipemia (49.6%), planting stents (81.9%), diabetes (31.4%) and smokers (47.8%). Additionally, many patients were taking proton pump inhibitors (PPI) (25.2%) and calcium channel blockers (CCB) (46.8%). Records revealed that 84 (9.9%) participants experienced cerebrovascular events, 94 (11%) participants underwent an acute myocardial infarction and 438 (51.5%) participants suffered unstable angina.

The S75N SNP genotype distributions for CC, CT and TT were 55.4% (n = 471), 43.7% (n = 372) and 0.2% (n = 2), respectively. Additionally, 0.7% (n = 6) patients failed genotyping. The allelic frequencies of the C and T alleles were 77.8% and 22.2%, respectively. The incidence of the C allele (11.4%, 12.6% and 55.8% in cerebrovascular events, acute myocardial infarction and unstable angina, respectively) was significantly higher than that of the T allele (4.8%, 6.1% and 37.5% in cerebrovascular events, acute myocardial infarction and unstable angina, respectively) (p < 0.001) among patients with endpoints. Additionally, the risk allele frequency of patients with endpoints was approximately half of that of patients without endpoints, as shown in Table 1. The CT and TT genotypes (CT + TT group) were combined in the subsequent analysis to elucidate the effect of the T allele (the dominant model for the T allele).

**Table 1:** The risk allele frequency of acute coronary syndrome patients in three outcome.

Clinical outcomes	Occurrence (N = 851)	N^§^(CC vs CT + TT) (N = 471 vs 374)	RAF	P*	OR^Ϯ^ (95CI)
Cerebrovascular event	YES (N = 84)	66 vs 18	0.11	1.521 × 10^−4^	0.39 (0.24–0.65)
	NO (N = 767)	405 vs 356	0.24		
Acute myocardial infarction	YES (N = 94)	71 vs 23	0.12	4.619 × 10^−4^	0.45 (0.29–0.71)
	NO (N = 757)	400 vs 351	0.24		
Unstable angina	YES (N = 439)	296 vs 141	0.16	3.948 × 10^−10^	0.48 (0.38–0.6)
	NO (N = 412)	175 vs 233	0.29		

^§^Numbers of CC genotype patients vs CT + TT genotype patients who occurred endpoints.

*The P value has been adjusted; CI = confidence interval; OR = odds ratio; RAF = risk allele frequency.

^Ϯ^Odds ratio represents the increase in the risk of happening endpoints for each copy of the risk allele compared with subjects who do not carry the risk allele. P values and odds ratios were estimated by PLINK.

### Clinical characteristics and outcomes in the S75N genotype groups

**Figure 1:**
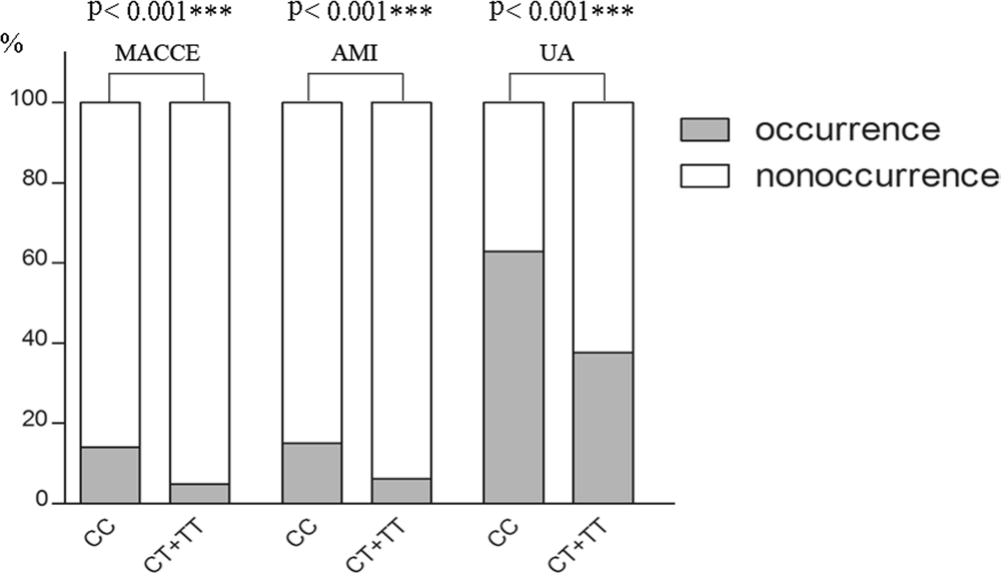
Occurrence ratios in the *CES1* S75N genotype group. Fourteen percent of CC group patients and 4.8% of CT + TT group patients had cerebrovascular events after clopidogrel treatment (*p* < 0.001). 15.1% vs 6.1% of patients suffered acute myocardial infarction and 62.8% vs 37.7% suffered unstable angina after treatment, respectively (*p* < 0.001). The total number of patients in the CC and CT + TT groups was 471 and 374, respectively.

The clinical factors, such as hypertension (*p* = 0.003), hyperlipemia (*p* < 0.001), stent (*p* = 0.003), CCB (*p* = 0.026) and PPI (*p* < 0.001), as well as the clinical outcomes of cerebrovascular event, acute myocardial infarction and unstable angina (p < 0.001) were significantly different between the CC and CT + TT genotype groups (Table 2). Figure 1 illustrates the occurrence ratios of three endpoints among the *CES1* S75N genotype groups. The occurrence ratios for each endpoint in the CC group were relatively higher for cerebrovascular events, acute myocardial infarction and unstable angina than that in the CT + TT group (14% vs 4.8%, 15.1% vs 6.1% and 62.8% vs 37.7, respectively). A logistic analysis with a CC group as a reference (odds ratio = 1.0) yielded an odds ratio of 0.31 (95% CI: 0.18 to 0.53), 0.37 (95% CI: 0.23 to 0.60) and 0.36 (95% CI: 0.27 to 0.47) for the likelihood of CT + TT genotype patients who had experienced cerebrovascular events, acute myocardial infarction and unstable angina, respectively.

**Table 2:** Clinical characteristics in the *CES1* S75N genotype groups in all subjects.

	CC (N = 471)	CT + TT (N = 374)	*P* value
Clinical characteristics			
Age (years)	62.86 ± 11.42	63.93 ± 11.87	0.185
Males	334 (70.9)	270 (72.2)	0.370
Smoking	224 (47.9)	181 (48.7)	0.437
Hypertension	345 (73.2)	240 (64.2)	0.003
Stent	401 (85.1)	290 (77.5)	0.003
Diabetes	159 (33.8)	108 (28.9)	0.075
Hyperlipemia	289 (61.4)	131 (35)	<0.001
CCB	235 (50.1)	161 (43.2)	0.026
PPI	86 (18.3)	126 (33.8)	<0.001
Clinical outcomes			
Cerebrovascular event	66 (14)	18 (4.8)	<0.001
Acute myocardial infarction	71 (15.1)	23 (6.1)	<0.001
Unstable angina	296 (62.8)	141 (37.7)	<0.001

Measure data are N (%), continuous data are mean ± standard deviation (SD), unless otherwise indicated.

### Association between genetic polymorphisms and three outcomes among acute coronary syndrome patients

**Figure 2:**
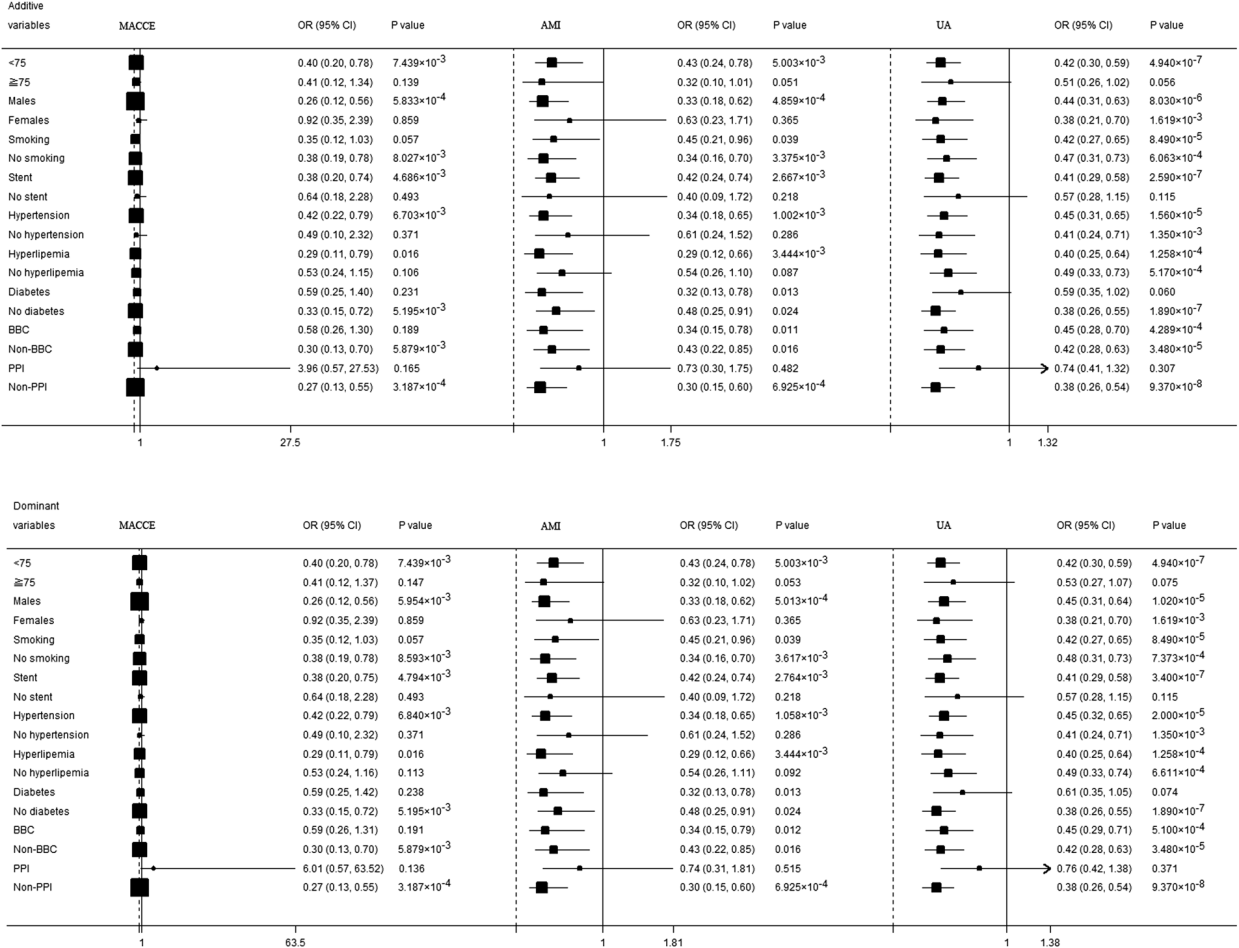
Association between genetic polymorphisms and clinical outcomes in acute coronary syndrome patients. Each box and horizontal line represents the odds ratio (OR) and 95% confidence interval (CI); each analysis used the C allele for reference. Eight factors were included in the analysis and all factors were adjusted for the other factors. For example, when analyzing age, the factors, including gender, smoke state, plant stent, hypertension, hyperlipemia, diabetes, BBC and PPI were adjusted.

To further explore the relationship between *CES1* S75N and the outcome of clopidogrel therapy, patients were stratified according to nine factors including age, gender, smoking state, stent, hypertension, hyperlipemia, diabetes, BBC and PPI, as shown in Fig. 2. In additive and dominant models, *CES1* S75N correlates with cerebrovascular events in ACS patients with any of the following qualities: <75 years old, male, no smoking, stent, hypertension, hyperlipemia, no diabetes, no BBC and no PPI. In additive and dominant models, *CES1* S75N also correlates with acute myocardial infarction in ACS patients with any of the following qualities: <75 years old, male, stent, hypertension, hyperlipemia, and no PPI. Smoking state, diabetes and BBC does not affect the relationship between S75N and acute myocardial infarction. Additionally, S75N also correlated to unstable angina in all ACS patients except those who took PPI and/or did not have a stent implantation in additive or dominant models, although a marginal significant difference existed in patients ≥75 and/or with diabetes.

## Discussion

A cohort of 851 Chinese patients with acute coronary syndrome was investigated to determine individual responses to clopidogrel. The rates of occurrence for cerebrovascular events, acute myocardial infarction and unstable angina were 9.9%, 11% and 51.5%, respectively. Our results showed that *CES1* S75N enhanced the clopidogrel efficacy, leading to a greater clopidogrel response. Moreover, the frequency of the T allele of rs2307240 in ACS patients was significantly higher than that in the general population.

Clopidogrel plays a key role in the treatment of ACS. Clopidogrel is biotransformed into 2-oxo-clopidogrel in hepatic tissue, followed by subsequent hydrolysis into its active thiol metabolite. This is a two-step oxidation reaction catalyzed by cytochrome P450 enzymes. Previous research reported that *CES1* was the major enzyme responsible for eliminating clopidogrel, 2-oxo-clopidogrel and the active metabolite^20, 21^. Additionally, recent investigations have shown that hydrolysis of clopidogrel by *CES1* leads to a more than 1,000-fold increase in plasma exposure of clopidogrel carboxylic acid compared to the exposure of the parent clopidogrel and its active metabolite^22^. Therefore, the production of inactive metabolites occurs at a considerably higher rate compared to the formation of the active metabolite^20^. Additionally, an increasing number of data suggest that the genetic variation which affects the activity and expression of CES1 may also play an important role in clinical treatment^23–25^.

The remarkably higher incidence of the C allele in the study endpoint group led us to hypothesize that this SNP has a physiological function. In theory, the occurrence of cerebrovascular events, acute myocardial infarction and unstable angina are higher in subjects with the CC genotype than those with the CT + TT genotype due to enhanced formation of active metabolites or reduced elimination of parent clopidogrel in the T allele carriers. The variation of *CES1* at position 75 results in a serine-to-asparagine substitution, which may influence protein phosphorylation and affect the catalytic function of CES1. S75N may alter the secondary structure of CES1 protein and influence the interaction between the CES1 protein and ligand. This finding resembles a previous result, in which the 143E substitution resulted in a complete loss of catalytic function^26^. Altermatively, this variation may also alter CES1 expression by affecting exon skipping, but requires further verification.

Genetic polymorphism plays an important role in the efficacy of clopidogrel treatment. However, clinical factors may influence treatment, such as age, gender, smoking state, stent, hypertension, hyperlipemia, diabetes, BBC and PPI. These variables have been eliminated by stratification analyses in our experiment. However, the association between *CES1* S75N and the clopidogrel response persisted after the stratification analysis. Additionally, the relationship between S75N and three clinical outcomes was evident in each subgroup. These results suggest that *CES1* S75N may independently affect clopidogrel therapy.

This is the first report on *CES1* S75N and it shows the effect of the *CES1* S75N variation on clopidogrel therapy. Our results provide important insight into the use of *CES1* genotypes in prescribing the most effective anti-platelet therapy. Given that the frequency of the T allele within the general population is relatively low (approximately 5%), we found a much higher incidence in the study endpoint group (11–16%) and in the nonoccurrence group (24–29%). These data indicate that the mutation is a risk factor for acute coronary syndrome. Further research is required to elucidate this hypothesis and demonstrate how this SNP affects clopidogrel therapy and leads to an improved clinical response.

## Methods

### Study populations

A large cohort of 851 Chinese ACS patients from the Shi-jing-shan Institute of Hypertension (n = 315), Zhengzhou Central Hospital (n = 286) and Xiangya Hospital (n = 250) were eligible for this study, during the period of February 2007 to October 2015. The clinical diagnosis was based on the 2007 ACC/AHA guideline for the diagnosis and treatment of ACS, including acute ST-segment elevation myocardial infarction (STEMI), acute non-ST-segment elevation myocardial infarction (NSTEMI) and unstable angina (UA). All patients took the antiplatelet drug (clopidogrel) at a dose of 75 mg/d for more than one year. Exclusion criteria included contraindications to clopidogrel, noncompliance for more than 12 months, simultaneous anticoagulant treatment and severe hepatic or renal dysfunction. After patients underwent clopidogrel treatment for at least 12 months, a follow-up was performed by clinical visits or telephone interviews.

End-point collection was completed by an independent group that was unaware of genotype information. The primary outcome was the composite of cerebrovascular events (MACCE) and acute myocardial infarction (AMI). The secondary outcome was unstable angina (UA). Patients’ clinical outcomes consisted of cerebrovascular events (MACCE), acute myocardial infarction (AMI) and unstable angina (UA). Among them, acute myocardial infarction included STEMI and NSTEMI, which are defined as recent ischemic symptoms with electrocardiographic abnormalities in the ST segment (depression or elevation of at least 0.1 mV) and a positive troponin concentration as defined locally. UA and recurrent angina (in the hospital) are defined similarly, although electrocardiographic changes are not required. Cerebrovascular events include cerebral hemorrhage and cerebral infarction, which are diagnosed as a focal neurological deficit that persist for more than 24 hours with ischemic or hemorrhage cerebral lesions confirmed by computed tomography (CT) or magnetic resonance imaging (MRI).

This research complied with the Declaration of Helsinki, approval to conduct the research was obtained from the Chinese Clinical Trial Registry, and all participants provided written informed consent (registration number: ChiCTR-OPN-15006260).

### Genotyping

Genomic DNA was extracted from peripheral blood leukocytes using standard procedures (Puregene DNA isolation kit, Merck Eurolab). The SNP that potentially affected CES1 activity was chosen by UCSC. As a result, *CES1* S75N (rs2307240) was investigated and genotyping was performed by Sequenom MassArray (Sequenom; BioMiao Biological Technology, Beijing, China). The genotype call rate of *CES1* S75N was 99.3%.

### Statistical analysis

SPSS 18.0 (SPSS, Inc., Somers, NY, USA) was used to calculate summary statistics, distributions, and frequencies in all patients. Three different outcomes, including cerebrovascular events, acute myocardial infarction and unstable angina, were used to investigate the response of clopidogrel. Chi-square tests and Student’s t-tests were used to determine the differences in sex, age, stent (percutaneous coronary intervention with stent implantation), hypertension and smoking among the genotypes. Continuous data are described as the mean ± standard deviation (SD) in the text and tables. Measurement data are expressed as numbers (percentage). The stratified analysis of 851 subjects was performed using PLINK and the association analyses were conducted using two models after adjusting for covariates via Chi-square tests. The additive model accounted for the additive effects of SNPs. Dominant models were tested for the minor allele with two pooled classes. For example, if A is a minor allele, then a is the major allele. The dominant model indicates a genotype of AA or Aa versus aa. The stratified analysis was performed by Stata software.
